# Recognizing the Importance of Public Health Mycology

**DOI:** 10.3390/life15020145

**Published:** 2025-01-22

**Authors:** Felix Bongomin

**Affiliations:** 1 Department of Medical Microbiology and Immunology, Faculty of Medicine, Gulu University, Gulu P. O. Box 166, Uganda; f.bongomin@gu.ac.ug or drbongomin@gmail.com; 2 Department of Internal Medicine, Gulu Regional Referral Hospital, Gulu P. O. Box 180, Uganda; 3 Manchester Fungal Infection Group, Division of Evolution, Infection, and Genomics, School of Biological Sciences, Faculty of Medicine, Biology and Health, The University of Manchester, Manchester M13 9PL, UK

## 1. Introduction

Fungal diseases are an emerging global public health concern, affecting high-, low-, and middle-income countries [1]. Globally, 6.5 million invasive fungal infections are recorded annually and about 2.5 million deaths are attributed to fungal diseases [2]. Fungal infections due to *Aspergillus*, *Candida*, *Pneumocystis*, and *Cryptococcus* are the leading causes of morbidity and mortality [2,3], especially among individuals with a weakened immune system due to advanced human immunodeficiency virus (HIV) disease, cancers, chemotherapy, diabetes mellitus, and other underlying disorders [1]. Over 2.1 million people develop invasive pulmonary aspergillosis (IPA) annually, with an 85.2% mortality rate. About 1.6 million people develop invasive candidiasis, and 63.6% die each year [2]. *Pneumocystis jirovecii* pneumonia (PCP) affects 505,000 individuals, with a 42.4% mortality rate, while cryptococcal meningitis affects 194,000 people, with a mortality rate of 75.8% [2]. Moreover, the increasing incidence, antifungal resistance, and geographical range of fungi infections has been linked to climate change [4,5,6].

**Figure 1:**
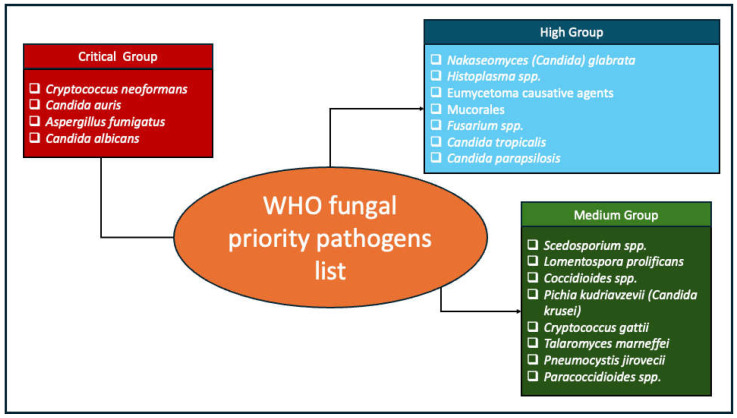
World Health Organization fungal priority pathogen list.

Recognizing the growing global health threat of fungal infections, the World Health Organization (WHO) developed its first fungal priority pathogens list in 2022 to guide research, development, and public health action [1], Figure 1. Several fungal pathogens including the WHO priority pathogens are increasingly becoming resistant to current antifungal drugs [3,7]. This is partly driven by inappropriate antifungal drug use across the One Health scope [1]. For instance, the use of agricultural fungicides is responsible for the rising rates of azole resistance *Aspergillus fumigatus* infections [7,8,9,10,11]. Further, access to quality antifungal drugs and diagnostic tests is limited, particularly in low-resource settings, where the fungal disease burden is the highest [12]. As such, many fungal infections remain undiagnosed and untreated [12].

The Global Action for Fungal Infection (GAFFI) has attempted to enumerate the burden of fungal disease in several countries across the world. However, empirical studies remain scarce, and continued surveillance for fungal diseases is key to determine the trends of these important causes of mortality and morbidity. This Special Issue includes original contributions and reviews of the current state of fungal diseases at the global level, and aims to suggest a way forward in the effort to curb the growing burden of fungal diseases.

### An Overview of Published Articles

Kang et al.’s article (Contribution 1) assessed the incidence and clinical characteristics of PCP in non-human immunodeficiency virus patients at a tertiary hospital between 2016 and 2022, with an aim of evaluating the impact of the coronavirus diseases-2019 (COVID-19) pandemic on PCP. The study revealed a significantly higher incidence of PCP in the COVID-19 era (37/1000 patient-years) as compared to the pre-COVID-19 era (13.1/1000 patient-years) (*p* < 0.001). Additionally, it showed a significant increase in PCP co-infection with IPA in the COVID-19 era (2.4% vs. 18.3%, *p* = 0.013). The authors also highlighted independent risk factors for PCP-related mortality including previous glucocorticoid use, hypoxemia, acute kidney injury, and IPA co-infection. They also identified risk factors for IPA in patients with PCP including previous use of tyrosine kinase inhibitors, COVID-19 within 30 days, leukopenia, and intensive care unit admission. Furthermore, they noted that 12 (16.9%) patients with PCP had a history of COVID-19 within 90 days; however, infection was not associated with mortality. The study concludes that active evaluation of patients with suspected PCP and assessment of IPA co-infection risk may help improve the outcomes of patients with PCP.

The article by Hsu et al. (Contribution 2) is a case summary and literature review on histoplasmosis in Taiwan. The authors summarized a total of 17 cases reported in Taiwan over the past 40 years and provided detailed descriptions for four probable indigenous histoplasmosis cases. However, they highlighted the lack of data on the environmental surveillance for the *H. capsulatum* complex in Taiwan as a limitation to their review. The main article conclusion points to the need to conduct further phylogenetic analysis on both environmental and clinical isolates to provide valuable evidence for the region.

In the third article, Kruithoff et al. (Contribution 3) focuses on dermatophyte infections. The authors highlight that antifungal resistance has led to the increase in the incidence of dermatophyte infections. Antifungal resistance was observed among the most common dermatophyte species such as *Trichophyton rubrum* and *Trichophyton mentagrophytes* and a new subspecies, known as *Trichophyton indotineae*. They emphasized the importance of conducting antifungal susceptibility testing to select the appropriate antifungal necessary for successful treatment.

Rozaliyani et al. (Contribution 4) described the fungal and bacterial microbiome interactions in the respiratory tract, with a focus on *Aspergillus* spp. This study showed that *Aspergillus* spp. colonies may induce various unfavorable inflammatory responses in the respiratory tract. However, bacterial microbiomes such as *Pseudomonas aeruginosa* were revealed to cause several mechanisms that inhibit or stimulate *Aspergillus* spp. life cycles.

The fifth article published in this Special Issue is a review by Caetano et al. (Contribution 5). Recognizing the important role of fungi in human health and in the stability of the microbiota, the authors described the role of yeasts in human health. They summarized current information about yeasts that inhabit the human body and some of the diseases that they cause when the microbiota becomes unstable. The authors discussed yeasts including the genus *Candida*, *Malassezia*, *Rhodotorula* and *Cryptococcus* because they inhabit various niches.

The sixth article is a scoping review by Ekeng et al. (Contribution 6) with an aim of comparing the clinical presentation of gastrointestinal histoplasmosis (GIH) in people with and without HIV infection. The authors conducted a literature search of published cases of GIH from 2001 to 2021 and found 212 cases. Most cases were from North America (n = 88, 41.5%) and South America (n = 79, 37.3%). They included 123 cases (58.0%) in both clinical and pathological analyses and excluded the remainder that did not have clinical and pathological findings. Of the 123 cases, 41 had HIV infection while 82 were without HIV infection. The diagnosis was made predominantly by histopathology (n = 109, 88.6%). A significant proportion of people with HIV infection had abdominal pain as the most predominant symptom of GIH compared to those without HIV infection (65.9% versus 41.9%, *p* < 0.05). The colon was the most affected site with a slightly higher proportion in those with HIV infection compared with cases without HIV infection (46.3% versus 42.7%). The most common pathologic findings were caecal and ileal ulcers. Caecal ulcers were significantly more frequent in cases with HIV infection compared to those without HIV (32.1% versus 7.1%, *p* < 0.05). The authors conclude that despite GIH being more common in people with HIV infection, it also affects people without HIV infection with similar clinical presentations.

Reyes-Montes et al.’s work (Contribution 7) is a review on the epidemiology, diagnosis and treatment of coccidioidomycosis cases in the Americas in the period of 1950 to 2021. The authors reviewed fifty-nine articles, corresponding to 275 clinical cases. The review showed a higher incidence of coccidioidomycosis in the male gender than the female gender. The most affected age group was 31–40 years, and the most reported clinical presentation was disseminated with greater involvement in cutaneous and subcutaneous tissue, followed by the CNS, bone system, and peritoneum. The species most frequently reported was *C. immitis*. The most used treatment was azoles, followed by their combination with amphotericin B, monotherapy with amphotericin B, and alternative medicine. The authors concluded that serological tests are the preferred diagnostic method in daily medical practice, and cultures remain the gold standard. Furthermore, ketoconazole and amphotericin B, individually or in combination, were highlighted as the treatment for coccidioidomycosis. However, they noted that epidemiological data outside the USA are still scarce.

## 2. Conclusions

Fungal diseases are an emerging public health crisis, yet they remain underrecognized despite their significant impact on global health, particularly in resource-limited settings. This Special Issue features the urgent need to prioritize public health mycology as a critical field to address the growing burden of fungal infections. The articles provide valuable insights into the epidemiology, clinical manifestations, and management of fungal diseases, highlighting the gaps in diagnostics, treatment, and surveillance that hinder effective control efforts.

Addressing these challenges requires a collaborative and coordinated approach. Public health entities such as the GAFFI, the Centers for Disease Control and Prevention (CDC), and the WHO have been instrumental in raising awareness and setting global priorities, including the development of the WHO fungal priority pathogens list. Moving forward, it is imperative to strengthen fungal disease surveillance, expand access to reliable diagnostics and antifungal therapies, and mitigate antifungal resistance through a One Health approach.

Investments in research, capacity building, and public health interventions will be pivotal in advancing public health mycology and reducing the burden of fungal diseases. By fostering partnerships among researchers, policymakers, and global health organizations, we can ensure that fungal diseases receive the attention they deserve and improve outcomes for affected populations worldwide.
